# A population pharmacokinetic model for creatinine with and without ingestion of a cooked meat meal

**DOI:** 10.1007/s00228-022-03398-9

**Published:** 2022-10-10

**Authors:** Zhendong Chen, Chunli Chen, Max Taubert, Michael Mayersohn, Uwe Fuhr

**Affiliations:** 1 Department I of Pharmacology, Center for Pharmacology, Faculty of Medicine and University Hospital Cologne, University of Cologne, Gleueler Straße 24, Cologne, 50931 Germany; 2 Heilongjiang Key Laboratory for Animal Disease Control and Pharmaceutical Development, College of Veterinary Medicine, Northeast Agricultural University, Changjiang Road 600, Xiangfang District, Harbin, 150030 People’s Republic of China; 3 College of Pharmacy, University of Arizona, Tucson, AZ USA

**Keywords:** Creatinine, Population pharmacokinetic model, Volume of distribution, Creatinine generation rate

To the Editor,

Commonly used equations for estimating creatinine clearance (CL) and/or glomerular filtration rate (GFR) from serum creatinine are based on steady-state assumptions regarding creatinine formation and elimination and are therefore less applicable to patients with unstable renal function [1–3]. A compartmental creatinine model that can describe the dynamics in creatinine parameters over time was previously introduced by Ullah et al. to estimate creatinine clearance [4]. In this model, volume of distribution (*V*_d_) of creatinine was fixed at 60% of total body weight, corresponding to total body water (TBW). This approximation ignores variability, which may lead to biased estimates of other pharmacokinetic (PK) parameters [4]. Therefore, there is a need to enrich the estimation of creatinine PK parameters with experimental data, in the case of *V*_d_ including exogenous administration of creatinine. Creatinine PK profiles in healthy subjects with and without ingestion of a cooked meat meal as a creatinine source were previously reported by Mayersohn et al. [5]. Data from this study were reanalyzed using a population pharmacokinetic (PPK) approach to estimate PK parameters for creatinine, refine the dynamic creatinine model, and reduce bias in the estimation of other kinetic parameters.

A one-compartment PK model with linear elimination, first-order absorption, and zero-order creatinine generation was constructed based on the dataset composed of 133 serial creatinine plasma concentrations and 11 creatinine amounts excreted in urine during 24 h. Exponential models were most suitable to describe inter-individual variability (IIV), while two separate proportional errors were best to describe residual variability of plasma concentrations and amounts excreted in urine, respectively. The individual bioavailable creatinine dose for each subject was estimated using a pre-defined arbitrary population dose (180 mg) multiplied by individual post hoc estimates for apparent bioavailability (F1). Both the goodness of fit and visual predictive check plots (Supplemental Figs. 1 and 2) and the bootstrap results showed that the final model was reliable and stable.

Table 1 lists the PK parameters estimated from the final model and the 95% confidence intervals derived from 994 successful bootstrap samples. The typical values for CL and *V*_d_ of creatinine were estimated to be 7.92 L/h and 53.9 L (73.8% of the total body weight), which were similar to the reported values [5–7]. Incorporating variability on CL and *V*_d_ did not significantly improve the model, which may be attributable to the relative homogeneity of the small population studied. As a comparison, when fixing the value of *V*_d_ to the estimated TBW, which is 43.8 L (60% of 73 kg), the OFV will increase by 3.230, which does not represent a significant difference, while values of other parameters do not change much. Deciding between various error models was not straightforward due to the limited number of data points, but the point estimates for pharmacokinetic parameters remained essentially unchanged regardless of the error model chosen and are therefore considered as reliable.

**Table 1:** Parameter estimates obtained from the final model and bootstrap statistics (*n* = 1000)

	**Final model**		**994 successful bootstrap runs**	
**Parameter**	**Estimates**	**RSE (%)**	**Median**	**95% CI**
Ka (1/h)	1.76	26.7	1.60	0.715–3.34
CL (L/h)	7.59	6.4	7.59	6.71–8.48
*V*_d_ (L)	53.9	20.0	53.0	31.2–72.0
F1	1.57	21.1	1.60	1.04–2.07
CGR (mg/h)	67.9	6.9	67.9	59.4–76.3
Lag time (h)	0.344	4.7	0.344	0.297–0.370
**IIV**				
Ka (CV%)	55.3 (1.6%)	37.7	55.4	3.8–83.1
F1 (CV%)	24.6 (0.1%)	20.3	24.0	15.0–30.0
CGR (CV%)	3.9 (0.1%)	25.8	3.5	1.1–4.7
**Residual variability**				
Proportional error, % (plasma)	3.9 (7.0)	10.0	3.8	3.2–4.5
Proportional error, % (urine)	15.5 (0.2)	21.8	14.7	6.4–19.7

For IIV, the corresponding shrinkage estimates are shown in parentheses

*Ka* apparent absorption rate constant, *CL* renal clearance, *V*_*d*_ the volume of distribution, *F1* dose correction factor, *CGR* creatinine generation rate

The typical value of the creatinine generation rate (CGR) in healthy volunteers was 68.0 mg/h, which is consistent with the value of 65.8 mg/h (male, 31 years, 73 kg) calculated based on the reported equation [2], but higher than the value of 42.8 mg/h and 43.8 mg/h in patients reported by Ullah et al. and Daugirdas et al., respectively [5, 8]. The estimated dose (293 ± 62.0 mg, *n* = 6) was not fully consistent with the increased creatinine amount excreted in urine after beef ingestion (180 ± 102 mg, *n* = 5). This does not exclude the possibility that these discrepancies are related to the accuracy of the raw data; furthermore, no demographic information is available in the publication to better define the PPK model.

In this evaluation, a PPK creatinine model was developed using creatinine data with and without ingestion of boiled beef in healthy volunteers. Reasonable parameter estimates including CGR and *V*_d_ were obtained from the final well-structured model. The model is a useful starting point for further experimental approaches to improve the understanding of creatinine kinetics, which may involve creatinine “dosing” accompanied by independent methods to assess GFR, e.g., by a test dose of iohexol.

## Supplementary Information

Below is the link to the electronic supplementary material.

Supplementary file1 (DOCX 137 KB)

## Data Availability

The data that support the findings of this study are available from the corresponding author upon reasonable request.
