# Supplementary material for: A population pharmacokinetic model for creatinine with and without ingestion of a cooked meat meal
Source: Eur J Clin Pharmacol. 2022 Oct 10;78(12):1945–7. doi: 10.1007/s00228-022-03398-9 (PMC9649480; doi:10.1007/s00228-022-03398-9)
Supplement: Supplementary file 1 — Supplementary file1 (DOCX 137 KB) [file 228_2022_3398_MOESM1_ESM.docx]

Supplementary figure 1 Combined goodness-of-fit plots of creatinine (A – D) plasma concentration and (E – H) urine excreted amount.

CWRES, conditional weighted residuals. A: observed *vs*. individual predicted plasma concentration; B: observed *vs*. population predicted plasma concentration; C: CWRES of plasma *vs*. time; D: CWRES of plasma *vs*. population predicted plasma concentration; E: observed *vs*. individual predicted amount excreted in urine; F: observed *vs*. population predicted amount excreted in urine; G: CWRES of urine *v*s. time; H: CWRES of urine *vs.* population predicted excreted amount.


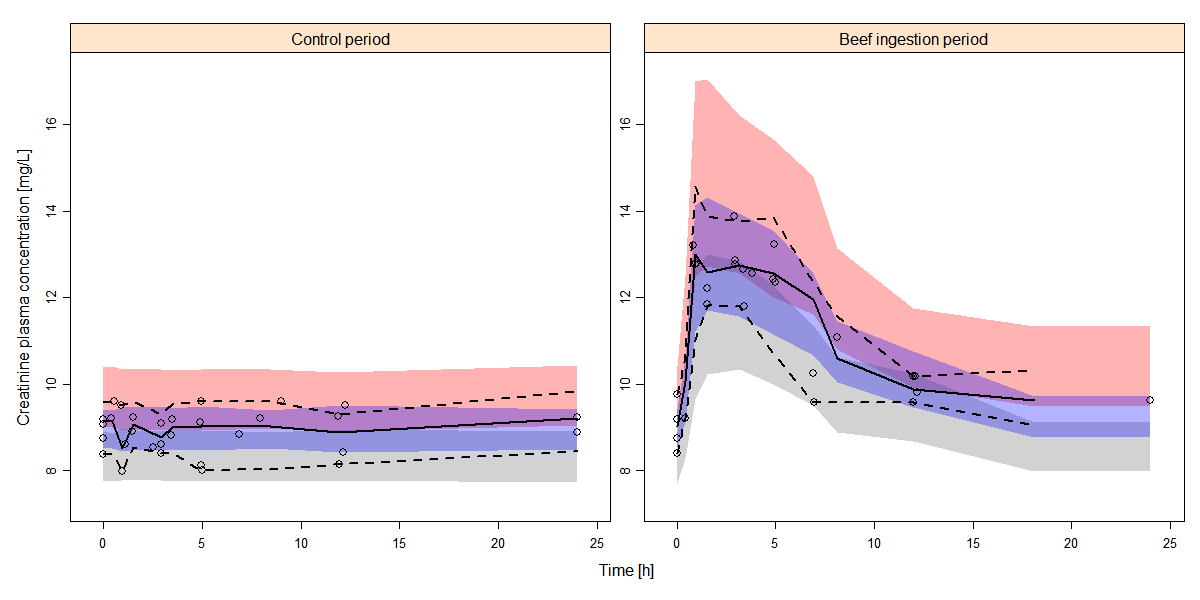
 Supplementary figure 2 Confidence interval visual predictive check (n = 1000) for the final model in plasma for the control period and beef ingestion period.

Dots represent observed concentrations. Black solid lines represent the median while dashed lines show the 5^th^ and 95^th^ percentile of observed concentrations. Shaded areas are the model-predicted 95% CIs for the 5^th^ (grey), 50^th^ (blue), and 95^th^ (red) percentiles from 1000 simulated datasets.
